# MicroRNA 107 Partly Inhibits Endothelial Progenitor Cells Differentiation via HIF-1β

**DOI:** 10.1371/journal.pone.0040323

**Published:** 2012-07-06

**Authors:** Shu Meng, JiaTian Cao, LianSheng Wang, Qing Zhou, YiGang Li, ChengXing Shen, XiaoPing Zhang, ChangQian Wang

**Affiliations:** 1 Department of Cardiology, School of Medicine, Shanghai Jiao-tong University, Xin-hua Hospital, Shanghai, China; 2 Department of Cardiology, School of Medicine, Shanghai Jiao-tong University, No. 9 People's Hospital, Shanghai, China; 3 Department of Laboratory Medicine, School of Medicine, Shanghai Jiao-tong University, No.9 People's Hospital, Shanghai, China; 4 Department of Nuclear Medicine, School of Medicine, Tongji University, Shanghai 10th People's Hospital, Shangha, China; Harvard Medical School, United States of America

## Abstract

Endothelial progenitor cells (EPCs) play an important role in tissue repair after ischemic heart disease. In particular, the recovery of endothelial function is reliant on the ability and rate of EPCs differentiate into mature endothelial cells. The present study evaluated the effect of microRNA 107 (miR-107) on the mechanism of EPCs differentiation. EPCs were isolated from rats' bone marrow and miR-107 expression of EPCs in hypoxic and normoxic conditions were measured by real-time qualitative PCR. CD31 was analyzed by flow cytometry and eNOS was examined by real-time qualitative PCR and western blotting and these were used as markers of EPC differentiation. In order to reveal the mechanism, we used miR107 inhibitor and lentiviral vector expressing a short hairpin RNA (shRNA) that targets miR-107 and hypoxia-inducible factor-1 β (HIF-1β) to alter miR107 and HIF-1β expression. MiR-107 expression were increased in EPCs under hypoxic conditions. Up-regulation of miR-107 partly suppressed the EPCs differentiation induced in hypoxia, while down-regulation of miR-107 promoted EPC differentiation. HIF-1β was the target. This study indicated that miR-107 was up-regulated in hypoxia to prevent EPCs differentiation via its target HIF-1β. The physiological mechanisms of miR-107 must be evaluated if it is to be used as a potential anti-ischemia therapeutic regime.

## Introduction

Ischemic heart disease is one of the most prominent health problems throughout the world and carries with it a high mortality rate. A complete understanding of the biological pathways that are integral in recovery is necessary to develop optimal therapeutic strategies. Circulating endothelial progenitor cells (EPCs) are mobilized from the bone marrow in response to tissue ischemia [1]. At sites of vessel injury, EPCs can differentiate into mature endothelial cells and are known to play critical role in tissue repair and endothelial function recovery [2], [3]. Despite the potential for the EPCs to promote recovery at the cellular level, cellular oxygen homoeostasis is central to the pathophysiology of ischemia. [4] In low oxygen environments, hypoxia-inducible factor-1α (HIF-1α), the major hypoxia-regulated transcription factor, is amplified and overexpressed [5], [6]. Under hypoxic condition, HIF-1α heterodimerizes with hypoxia-inducible factor-1 β (HIF-1β), which is another subunit of HIF-1. HIF-1α then translocates to the nucleus where the HIF-1 complex binds to the hypoxia-response element (HRE) and activates the expression of target genes implicated in cell growth and survival. [7] Our previous research indicated that overexpression of HIF-1α would promote the differentiation of EPCs ex vivo [8]. We also found that HIF-1α knockdown via adenoviral small interfering RNA (siRNA) transfer inhibited EPC differentiation [9]. However, it is still unknown how HIF-1β, which heterodimerized with HIF-1α, acts during EPC differentiation in hypoxic conditions.

A functional link exists between hypoxia and microRNAs (miRs). Microarray-based expression profiles revealed miR-107 was induced in response to low oxygen conditions [6]. Although this provides evidence of a relationship between hypoxia and miRs, several questions still remain. First, it is still unknown whether hypoxia increases miR-107 expression in EPCs via a HIF dependent mechanism. Second, the effect of miR-107 overexpression on EPC differentiation is still unknown. Finally, there is no evidence as to which subunit of HIF, α or β, should be the target of miR-107 during its action on EPCs. Therefore the purpose of this study was to establish a lentivirus transduction protocol that allows highly efficient transduction of EPCs using the green fluorescence protein (GFP) gene as a marker. Subsequently, we aimed to determine the potency and the mechanism of miR-107 or shRNA-miR-107 on EPC differentiation.

## Methods

### Isolation and identification of EPCs

All procedures were approved by Institutional Animal Care and Use Committee of JiaoTong University Shanghai Medical College and conformed with US National Institutes of Health or European Commission guidelines. Eight weeks old Male Lewis rats were bought from Shanghai Slac Laboratory Animal Co LTD. The rats were injected and sacrificed with over dose of sodium pentobarbital and the femurs and tibias were removed from the rats. The bone marrow cavities were flashed with 0.01 mol/L precooled phosphate-buffered saline (PBS). The cellular pellets were washed with PBS and resuspended in M199 medium (Gibco). Bone marrow mononuclear cells (BMMCs) were isolated from the cell suspension by centrifugation through a Ficoll-isopaque (Sigma) density gradient. To obtain the EPCs [5], the BMMCs were allowed to adhere to 6-well plates in M199 medium for 1 h at 37°C in a 5% CO_2_ incubator. Then, the non-adherent cells were collected and cultured in M199 medium supplemented with 10% fetal calf serum (Hyclone, Logan, UT), 10 ng/ml vascular endothelial growth factor (VEGF, Peprotech) and 2 ng/ml basic fibroblast growth factor (bFGF, PeproTech) at 37°C in a 5% CO_2_ incubator. After 3 h, non-adherent cells were removed. The adherent cells were collected and cultured for 7 days.

After 7 days in culture, the cells were incubated with ﬂuorescein isothiocyanate conjugated lectin from Ulex europeus agglutinin 1 (FITC-UEA-1; Sigma Deisenhofen, Germany) and 1,19-dioctadecyl-3,3,3939 -tetramethylindocar-bocyanine perchlorate (Dil)-labeled acetylated low density lipoprotein (Dil-ac-LDL) as previously described [7]. Incorporation of Dil Ac-LDL and binding of FITC-UEA-1 were detected with a confocal microscope (Leica Microsystems GmbH). Dual-stained cells positive for both LDL-ac-DiI and UEA-1 were identified as EPCs. The purity of the EPCs was analyzed by flow cytometry (FCM) after staining with PE-anti-CD34 (BD), PE-anti-KDR (CD309, BD) and PE-anti-CD133 (Miltenyi Biotec) [7].

### The differentiation of EPCs to endothelial cells

EPCs were induced into endothelial cells under a hypoxic condition. EPCs were cultured with 150 μmol/L cobalt chloride (CoCl_2_) and in the incubator (healforce) ﬂushed with a mixture of 1% O_2_, 5% CO_2_, and 94% N_2_ at 37 C. Cells were harvested after treatment at three different time periods (6, 10, or 14 days). The EPCs under the normal oxidative condition were cultured in incubator and flushed with a mixture of 20% O_2_, 5% CO_2_, and 75% N_2_ at 37°C. The expression of CD31 of EPCs was analyzed with FCM [8], and the expression of endothelial nitric oxide synthase (eNOS) was examined by real-time qualitative PCR and western blotting.

### Western blotting analysis

EPCs were obtained in different groups at different time points. EPCs were incubated with 400 ul lysis buffer containing 100 mM Phenylmethanesulfonyl fluoride (PMSF). Thirty micrograms of proteins were loaded onto 10% SDS-polyacrylamide gels and transferred onto PVDF membranes (Millipore, USA). Western blots were performed using primary antibodies for GAPDH antibody (1∶500 dilution in TBST), HIF-1β antibody or eNOS antibody, and secondary antibodies goat anti-rabbit (1∶4000 dilution) as previously described [9]. The autoradiographies were scanned and semiquantitatively analyzed.

### RNA extraction and real-time qualitative PCR

Total RNA was isolated from EPCs using the Trizol reagent according to the manufacturer's instruction (Qiagen kit). RNA was reverse transcribed using the RevertAid™ First Strand cDNA Synthesis Kits (Fermentas) and stem-loop RT primer as previously described [10]. Expression levels of the RNA (microrRNA-107, eNOS) were measured using TaqMan real-time PCR and the reaction was performed using a Roche LightCycler 480 system (Roche Diagnostics GmbH, Mannheim, Germany). All reactions were performed in triplicates. U6 and GAPDH RNA were used as an endogenous control.

### Anti-miRNA transfection

To down-regulate the expression of miR-107, miR-107 inhibitor (Ambion, AM17000) was transfected into EPCs using Lipofectamine 2000 (Invitrogen, Carlsbad, CA). The inhibitor was diluted in the Opti-MEM medium (Gibco) at a final concentration of 140 nM. To confirm the efficiency of transfection, the same amount of negative control anti-miRNA inhibitor (Ambion, AM17010) was also transfected. Then, cells were incubated for 36 h after which the differentiation of the EPCs was evaluated.

### Construction and transfection of plasmid and Lentiviral vector

The expression plasmids for hsa-miR-107 and HIF-1β were created [11]. The target sequence was amplified with primers of miR-107 (Table 1A) and HIF-1β (Table 1B) and cloned into sites (BamHI/EcoRI) of expression vector pCDH-CMV-MCS-EF1-Puro (System Biosciences, Mountain View, CA, USA). In order to down-regulate HIF-1β expression in EPCs, small hairpin RNAs (shRNAs) targeting EPCs' HIF-1β were generated. The effective scramble sequences are shown in Table 1C.

**Table 1:** The primers of target sequences for experiments.

A	miR- 107	forward	5'- AAAGAATTCCTGTTTCACTCGCCAAGC-3'
		reverse	5'-AAAGGATCCAGCGAGTGAGGAGGGAGA-3'
B	HIF-1β	forward	5'-AAAGAATTCATGGCGGCGACTACAGC-3'
		reverse	5'- AAAGGATCCCTATTCAGAAAAAGGGGGA -3'
C		forward	5'-*CCGG* **CCTGTTTCCATGAATAGACTG** *CTC*
	HIF-1β		*GAG* **CAGTCTATTCATGGAAACAGG** *TTTTTG*-3'
	shRNAs	reverse	5'-*AATTCAAAAA* **CCTGTTTCCATGAATAGACTG**
			*CTCGAG* **CAGTCTATTCATGGAAACAGG**-3'.

* In section C, the nucleotide forward or reverse oligo are in bolded and the stem loop sequences are underlined.

The scramble sequences were cloned into sites of (Age I/EcoRI) expression vector pLKO-shRNA1(sigma). All constructions were verified by sequencing. Lentiviral supernatants were produced by expression vector pCDH-CMV-MCS-EF1-Puro-miR107 co-transfection of 293 T cells (ATCC) with psPAX2, pMD2G and either pCDH-CMV-MCS-EF1-Puro- HIF-1β and pLKO-shRNA1- HIF-1β. Lentiviral vectors expressing miR107 (lenti miR-107), HIF-1β (lenti HIF-1β) and scrambled sequences of HIF-1β were generated. EPCs were cultured in 6-well plates (5×10^5^ cells each well) infected with lentiviral vectors at a multiple of infection (MOI) of 10, and expression of GFP was analyzed by FACS.

### Statistical analysis

All data are presented as mean ± standard deviation. Statistical analysis was performed using a 2-way ANOVA and two-tailed paired Student's t test where appropriate. P-values of <0.05 were considered statistically significant. All experiments were performed at least three times.

## Results

### Identification of EPCs

**Figure 1:**
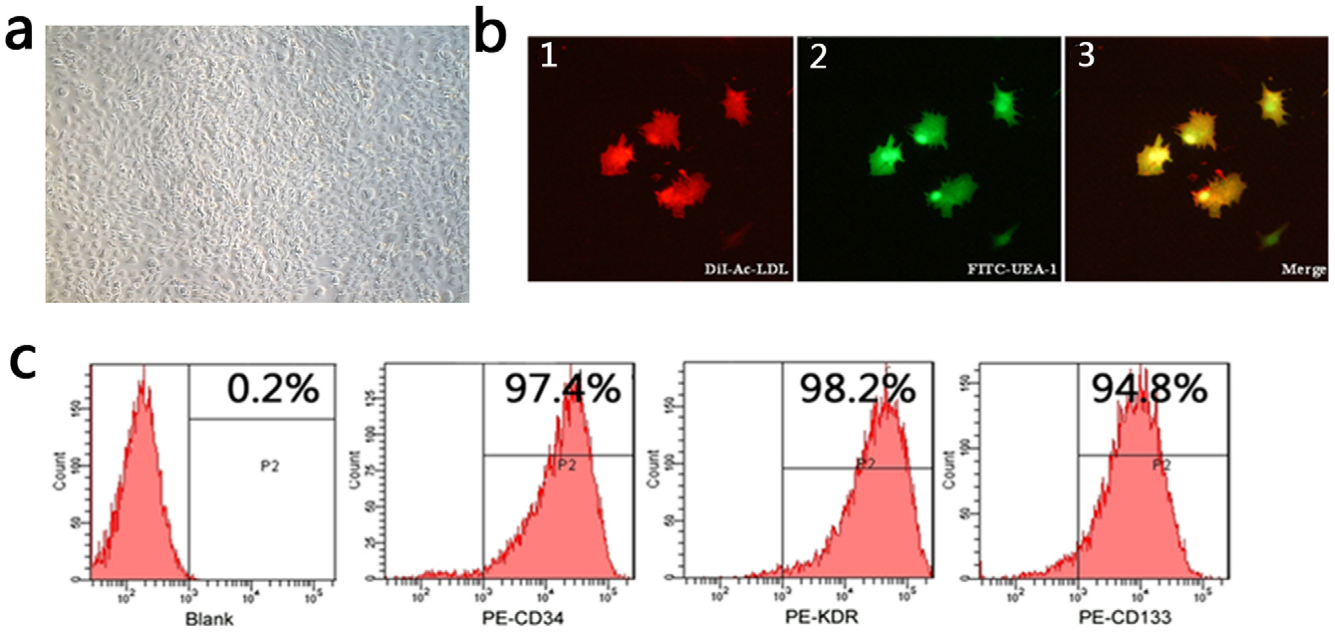
Identification of EPCs by co-stained with FITC-UEA-1 and Dil-ac-LDL and Flow cytometric analysis. a) Microscopic image of EPCs (200×). After 7 days of isolation, two different types of EPCs were found with most being spindle-shaped cells and a few presenting as polymorph cells. b) Incorporation of Dil-Ac-LDL (1) and binding of FITC-UEA-1 (2) were detected with a confocal microscope. The merged image (3) represents Dil-Ac-LDL+FITC-UEA-1+ cells. Dual-positive stained cells were identified as EPCs. c) Flow cytometric analysis of the cell surface markers indicated the expression profile of EPCs included CD34 (97.4%), KDR (98.2%) and CD133 (94.8%) cells.

The EPCs originally isolated from BMMCs were round and small. After 3 days in culture, cell clusters appeared. In 6–7 days a number of spindle-shaped cells sprouted from the edges of the cell cluster. Then cells gradually became bigger and appeared as into a cobblestone-like structure (Fig. 1a). At day 7, adherent cells were identified by co-stained with FITC-UEA-1 and Dil-ac-LDL. We observed double-positive cells by confocal scanning microscopy (Fig. 1b). In addition, FACS analyses were performed to confirm the endothelial phenotype of the EPCs, including CD34, KDR and CD133 (Fig. 1c).

### MicroRNA 107 expression and EPC differentiation under hypoxic condition

**Figure 2:**
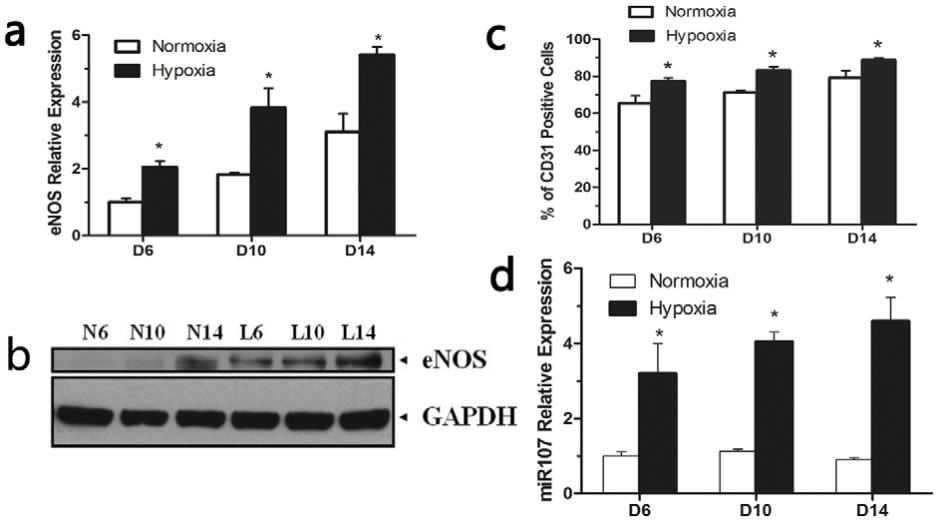
Hypoxia promoted EPC differentiation and increased miR-107 expression. The expression of eNOS was detected by Q-PCR (a) and western blotting (b). At the 3 time points, the expression of eNOS was higher under hypoxic conditions than that under normoxic conditions. c) Flow cytometric analysis indicated the expression of CD31 increased in the hypoxia group. d) miR-107 expression under normoxic and hypoxic conditions were detected by Q-PCR. The miR-107 was highly expressed under the hypoxic condition. (*P<0.05 vs normoxia group at days 6, 10 and 14). [The EPCs cultured under normoxic condition at 6, 10 and 14 days marked are as N6, N10, N14. The EPCs cultured under hypoxic conditions at 6, 10 and 14 days are marked as L6, L10, L14.].

To explore the biological function of miR-107, we first defined its expression in endothelial cells under hypoxic conditions. After the EPCs were harvested, they were cultured for 6, 10 and 14 days. We found miR-107 was highly expressed under hypoxic conditions (Fig. 2d). To evaluate the EPCs differentiation under hypoxic conditions, the expression of CD31 and eNOS were detected. This is a marker of EPCs differentiation into endothelial cells. The expression of CD31 was higher than the normal oxidative condition at 3 time points (Fig. 2c). The hypoxic condition also up-regulated the eNOS at the mRNA and protein levels (Fig. 2a and b). Therefore, hypoxia promoted EPCs differentiation and increased miR-107 expression.

### Effect of miR107 on EPCs differentiation in vitro

**Figure 3:**
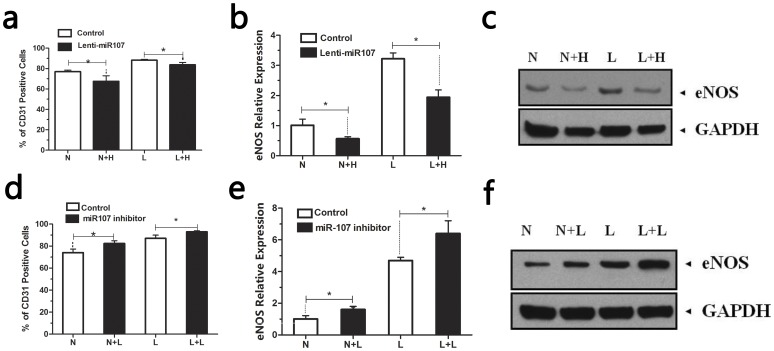
MiR-107 suppressed the differentiation of EPCs. (**1**) Upregulation of miR-107 suppressed the differentiation of EPCs.CD31 (a) and eNOS (b and c) expression of EPCs were determined after up-regulation of miR107. (*p<0.05 between groups). [N: EPCs cultured under normoxic conditions; L: EPCs cultured under hypoxic conditions; N+H: EPCs infected with Lentiviral vector expressing miR107 and cultured under normoxic conditions; L+H: EPCs infected with Lentiviral vector expressing miR107 and cultured under hypoxic conditions.] (**2**) Downregulation of miR-107 promoted the differentiation of EPCs.CD31 (d) and eNOS (e and f) expression of EPCs were determined after inhibition of miR107. [N: EPCs cultured under normoxic conditions; L: EPCs cultured under hypoxic conditions; N+L: EPCs transfected with miR-107 inhibitor and cultured under normoxic conditions; L+L: EPCs transfected with miR-107 inhibitor and cultured under hypoxic conditions. (*p<0.05 between groups)].

To explore the role of miR-107 in the EPC differentiation in vitro, we altered the endogenous miR-107 in the EPCs. The EPCs were infected by Lentiviral vector expressing miR-107 to up-regulate miR-107. Then, miR-107 over-expressed EPCs and control group were induced into endothelial cells under hypoxic or normoxic conditions. After 14 days, CD31 and eNOS were detected as previously described to determine EPC differentiation (Fig3 a, b and c). These data suggest the overexpression of miR-107 in EPCs suppressed the differentiation of EPCs. We next transfected EPCs with miR-107 inhibitor. After 14 days, the analysis of the differentiation revealed that the down-regulation of miR-107 promoted the differentiation of EPCs (Fig 3 d, e and f).

### The effects of miR-107 on HIF-1β expression in normoxia and hypoxia

**Figure 4:**
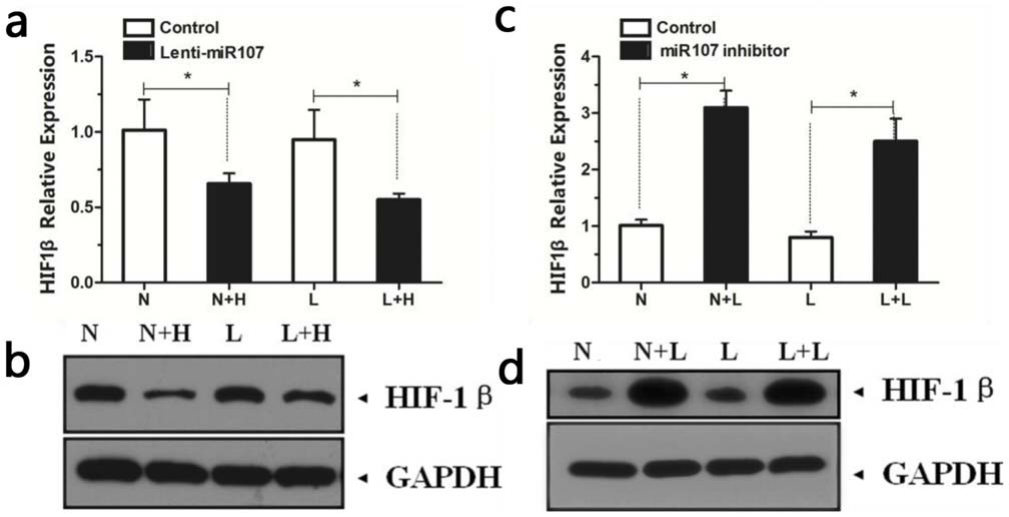
MiR-107 decreased HIF-1β expression of EPCs in normoxia and hypoxia. The HIF-1β expression of the EPCs were tested by Q-PCR (a and c) and western blotting (b and d) after the up-regulation of miR-107 (a and b) and inhibition of miR-107 (c and d). Overexpression of miR-107 inhibited mRNA and protein expression of HIF-1β (a and b), miR-107 inhibitor increased HIF-1β expression (c and d) (*p<0.05 between groups). [N: EPCs cultured under normoxic conditions; L: EPCs cultured under hypoxic conditions; N+H: EPCs infected with Lentiviral vector expressing miR-107 and cultured under normoxic conditions; L+H: EPCs infected with Lentiviral vector expressing miR-107 and cultured under hypoxic conditions; N+L: EPCs transfected with miR-107 inhibitor and cultured under normoxic conditions; L+L: EPCs transfected with miR-107 inhibitor and cultured under hypoxic conditions.].

The HIFs played the important role in EPC differentiation. The effect of microRNA 107 on the expression of HIF-1β was evaluated. We found that up-regulation of miR-107 in EPCs substantially decreased HIF-1β expression (Fig 4 a and b), and vice versa (Fig 4 c and d).

### MiR107 suppress EPCs differentiation via down-regulation of HIF-1β

**Figure 5:**
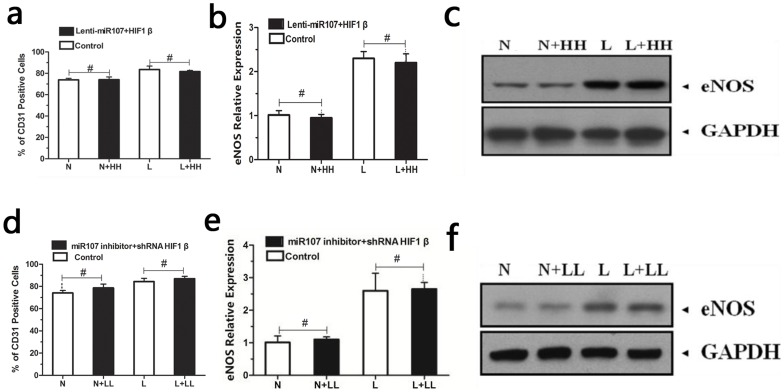
MiR107 suppress EPCs differentiation via down-regulation of HIF-1β. CD31 (a) and eNOS (b and c) expression of EPCs after infection by both lenti miR-107 and lenti HIF-1β (#p>0.05 between groups). CD31 (d) and eNOS (e and f) expression of EPCs after infection by siRNA against lenti miR-107 and HIF-1β (#p>0.05 between groups). There were no significant changes in the control and lentivirus infected groups. [N: EPCs cultured under normoxic conditions; N+L: EPCs infected with both lenti miR-107 and lenti HIF-1β cultured under normoxic conditions; L: EPCs cultured under hypoxic conditions; L+L: EPCs infected with both lenti miR-107 and lenti HIF-1β cultured under hypoxic conditions. N+HH: EPCs infected with lentivirus carrying siRNA against both miR-107 inhibitor and HIF-1β cultured under normoxic conditions; L+HH: EPCs infected with lentivirus carrying siRNA against both miR-107 inhibitor and HIF-1β cultured under hypoxic conditions.].

To investigate the correlation between miR-107 and HIF-1β on EPCs differentiation, we altered their endogenous expression. Both lenti-miR107 and lenti-HIF-1β were transfected into EPCs. EPCs overexpression of miR-107 and HIF-1β were cultured in hypoxic or normoxic conditions for 14 days. When compared to the negative control, eNOS did not significantly change. This is because lenti- HIF-1β increased endogenous HIF-1β expression, while lenti-miR107 reduced miR-107 direct target HIF-1β expression in EPCs. Down-regulation of CD31 and eNOS after up-regulation of miR-107 was offset by up-regulation of HIF-1β (Fig.5 a, b, c), which showed the up-regulation of miR-107 would inhibit EPCs differentiation via inhibiting HIF-1β. In contrast, we also transfected EPCs with two lentiviruses, one carrying siRNA against miR-107 and another carrying siRNA against HIF-1β. We found that down-regulation of miR-107 up-regulated CD31 and eNOS, which was offset by down-regulation of HIF-1β in hypoxic and normoxic conditions (Fig 5 d, e, f). From this it can be concluded that down-regulation of miR-107 would promote EPCs differentiation via up-regulating HIF-1β.

## Discussion

Previous studies have shown that overexpression of HIF-1α promotes the differentiation of EPCs ex vivo [12], while HIF-1α knockdown via adenoviral siRNA inhibits EPCs differentiation [13]. However, it was unknown whether miR-107, the miR induced in response to low oxygen, would be overexpressed in hypoxic EPCs and subsequently affect the differentiation capacity of EPCs. Therefore, the aim of this study was to examine the expression of miR-107 in EPCs in hypoxia, and elucidate the effect and the mechanism of miR-107 on EPC differentiation. The results from the present study have revealed that 1) miR-107 expression were increased in EPCs under hypoxic conditions; 2) up-regulation of miR-107 partly suppressed the EPCs differentiation induced in hypoxia, while down-regulation of miR-107 promoted EPCs differentiation; and 3) HIF-1β was the target for this pathway. From these collective results we can conclude that miR-107 was up-regulated in hypoxic conditions to prevent EPC differentiation via its target HIF-1β.

In response to hypoxic conditions, mammalian cells mount a well-orchestrated response that includes specific changes in the collective of expressed proteins. The altered gene expression program allows the cell to respond to hypoxic conditions, triggering adaptive processes that include changes in cell division, survival, motility, or differentiation. During the past 5 years, multiple groups have reported specific alterations in miR profiles, which are ∼22-nt long ncRNAs associated with cellular mRNAs and typically repress gene expression by reducing their half-life and/or inhibiting their translation [14], upon hypoxic stress [15], [16], [17]. Collectively, these studies have identified over 50 miRs that are either up-regulated or down-regulated in low oxygen conditions; however, most of these miRs appear to be cell type specific. The present study revealed miR-107 expression increases in EPCs under hypoxic conditions.

miR-107 is overexpressed in several tumor types, including colon, pancreas, and stomach cancers [18]. A recent study has also shown that breast cancer patients with higher miR-107 levels display a significantly better probability of metastasis-free survival [19]. Our findings indicate that miR-107 expression increases in EPCs and partly inhibits their differentiation in hypoxic conditions. In hypoxic conditions, we found the degree of EPC differentiation exceeded that of miR-107 overexpressed EPCs. This result suggests that low oxygen alone induces EPC differentiation, while the addition of miR-107 partially inhibits EPC differentiation. Therefore, miR-107 may partly inhibit hypoxia induced HIF-1α overexpression and subsequently inhibit overexpression of HIF-1α induced EPC differentiation in hypoxic conditions. Conversely, knockdown of miR-107 in hypoxia induced EPC differentiation more than hypoxia alone.

There has been some controversy surrounding the target of miR-107. Some research using target prediction software has suggested that miR-107 targets 3′UTR of yak HIF-1α. These researchers stated that liver and blood specific stability of HIF-1α mRNA was regulated by miR-107 [20]. Contrarily, other researchers have reported that the 3′UTR of HIF-1β contained a potential binding element for miR-107 with an 8-nt match to the miR-107 seed region, suggesting that the HIF-1β 3′UTR is a target of miR-107 [21]. To identify the HIF-1α or β dependent pathways that are involved in miR-107 mediated EPC differentiation in hypoxia, we first explored miR-107 regulation of HIF-1α or β in EPCs under both hypoxic and normoxic conditions. We demonstrated that the overexpression of miR-107 in EPCs would down-regulate the expression of mRNA or the HIF-1β protein. Conversely, knockdown of miR-107 in EPCs would up-regulate HIF-1β expression. However, miR-107 did not affect the counterpart of HIF-1β, namely HIF-1α. Then we explored the relationship between miR-107 and HIF-1β in both normoxic and hypoxic conditions. When we overexpressed both miR-107 and HIF-1β in EPCs, the miR-107-inhibited EPC differentiation was counteracted. The same results occurred with knockouts of both miR-107 and HIF-1β in EPCs. Down-regulation of miR-107 inducing EPC differentiation was offset. Collectively, these findings revealed that HIF-1β is the target of miR-107 in EPCs. Overexpression of miR-107 in EPCs prevents their differentiation via down-regulation of HIF-1β, while under expression of miR-107 in EPCs induces their differentiation via up-regulation of HIF-1β.

The PI3K/Akt pathway is also known to be crucial for EPC differentiation [9], [22] and is activated by hypoxia in certain cell types [23], [24]. It is also known that interference with Akt reduces the accumulation of HIF in response to hypoxia [25]. Therefore it is possible that PI3K/Akt pathway inhibition may be the mechanism that regulates miR-107 modulated EPC differentiation, however, further research both *in vitro* and *in vivo* must be performed to validate this hypothesis.

miR-107 modulation of EPC differentiation may play an important role in angiogenesis because of its target, HIF-1β. Recent studies have shown that HIF-1β −/− knockout mice died in utero between 9.5 and 10.5 days of gestation due to the abnormalities of HIF-1β's known role in hypoxic induction of angiogenesis [26]. Additionally it was found that HIF-1β −/− embryonic stem cells failed to activate genes that normally respond to low oxygen tension [27]. Despite these initial investigations of HIF-1β, much has yet to be assessed with respect to its role in hypoxic conditions. One paper showed that HIF-1β is present in excess in normoxia [28], but failed to determine why. Weir et al [29]. demonstrated that HIF-1β expression was unchanged during acute hypoxia, but was down-regulated in prolonged hypoxia, although its mRNA remained unchanged. He concluded that this was due to the post- translational down-regulation of HIF-1β. Our present study agrees with these findings that HIF-1β is down-regulated post-transcriptionally by miR-107. Since our study suggests miR-107 is a negative feedback to hypoxia and HIF-1β is a target of miR-107, we believe that HIF-1β may be present in excess in normoxia in order to functionally decrease during the period at which miR-107 is up-regulated during hypoxic conditions. This would meet the requirements of HIF-1α whose mRNA or protein levels increases exponentially in low oxygen.

In conclusion, the present study indicated that miR-107 is up-regulated in hypoxia to prevent EPC differentiation via its target HIF-1β. There is a need to further evaluate the physiological mechanism of miR-107 if it is to be used as an anti-ischemia therapeutic regime.
